# Silk as a Dressing

**Published:** 1890-02-08

**Authors:** 


### SILK AS A DRESSING.

Dr. v. Giacich, of Vienna, has had prepared for surgical purposes a fine, white silk paper and silk charpie, either of which may be impregnated with antiseptics. They are said to have given much satisfaction, especially from their great power of absorption. The idea was suggested by the late Dr. Grossich, but a company has now been formed with patent rights throughout Europe and America.

